# Medicinal Plants for Overcoming Drug Resistance in Cervical Cancer

**DOI:** 10.3390/biology15020191

**Published:** 2026-01-20

**Authors:** Thabang Patience Marema, Kagiso Laka, Zukile Mbita

**Affiliations:** Department of Biochemistry, Microbiology & Biotechnology, University of Limpopo, Private Bag X 1106, Sovenga 0727, South Africa; thabsmarema@gmail.com (T.P.M.); kagiso.laka@ul.ac.za (K.L.)

**Keywords:** cervical cancer, chemotherapy, drug resistance, medicinal plants, bioavailability

## Abstract

Drug resistance remains a significant challenge in cancer therapy, accounting for most relapses and contributing substantially to cancer-related mortality worldwide. Several molecular processes are linked to the development of resistance to anticancer drugs, with the most studied mechanisms including epigenetic changes, drug efflux, cell survival signalling pathways, and inactivation of anticancer drugs. Both intrinsic and acquired forms of resistance hinder tumour cell elimination, reducing treatment success. This translates to poorer patient outcomes and the need for more aggressive treatment regimens. Therefore, understanding these molecular processes is crucial for enhancing the efficacy of anticancer therapy. Medicinal plants offer potential to counter various resistance mechanisms through their diverse phytocompounds. These compounds may offer benefits including consistent availability, anticancer potency, few side effects, and minimal drug resistance. However, the bioavailability of these phytochemicals and the lack of extensive clinical trials remain key challenges. Therefore, this review provides in-depth information on the mechanisms that lead to drug resistance during cervical cancer therapy, the challenges related to phytochemical bioavailability, the current status, and future needs for clinical trials evaluating the application of medicinal plants to combat drug resistance in cancer cells.

## Simple Summary

Cervical cancer treatment is often complicated by drug resistance, which limits the success of chemotherapy and contributes to poor patient outcomes. Medicinal plants have been investigated as potential therapeutic options to enhance drug responsiveness and reduce resistance. Recent preclinical evidence indicates that plant-derived compounds may improve treatment efficacy and provide alternative strategies for patients with limited treatment options. Although these approaches are promising, challenges such as poor bioavailability and limited clinical trial data remain. Addressing these gaps is essential for translating laboratory findings into meaningful clinical benefit.

## 1. Introduction

Cervical cancer is a prevalent gynaecological malignancy, forming inside the epithelium of the uterine cervix. An estimation of around 604,127 new cases and 341,831 fatalities attributed to the disease were reported in 2020, with nearly 90% of them arising in low- and middle-income countries. This high prevalence in these regions is largely driven by inadequate access to advanced healthcare services and late diagnosis, which contribute to increased mortality among underprivileged women [1,2]. Cervical cancer ranks fourth in global female cancer prevalence and is a leading cause of death [3]. Drug resistance is a major obstacle in cervical cancer management [4]. It drives relapse and contributes to rising mortality by reducing tumour sensitivity to antineoplastic drugs. As tumour cells acquire resistance, drug potency declines, leading to poor therapeutic responses and eventual treatment failure [5,6]. Drug resistance in cancer is remarkably similar to that of infectious illnesses, in that both complications are confronted by several rapidly expanding intrinsic and/or extrinsic factors [7].

The development of multidrug resistance is attributed to mutations and metabolic abnormalities that accumulate over time in tumours initially sensitive to chemotherapy [8]. Chemotherapy employs drugs, such as cisplatin, paclitaxel, and others, to inhibit the growth of malignant tumours and induce their eradication. The initial chemotherapeutic intervention in cervical cancer patients frequently demonstrates significant efficacy; however, as treatment progresses, drug resistance often develops in most patients, thereby constraining the overall effectiveness of the therapy [5,9].

Over the last few decades, medicinal plants have been extensively studied for cancer treatment, offering an alternative to conventional treatments with fewer adverse effects. Phytochemicals, which are secondary metabolites found in medicinal plants, may offer an alternative treatment strategy to combat drug resistance [10]. Moreover, they offer a viable therapy option for patients with multidrug resistance cancers by targeting multiple molecular processes, including blocking ABC transporters, sensitisation of cancer cells, induction of apoptosis, and modulation of pathways underlying multidrug resistance [11].

## 2. Methodology

### 2.1. Review Design

This study was conducted as a systematic review in accordance with the PRISMA 2020 guidelines to ensure transparency and reproducibility.

### 2.2. Search Strategy

A systematic literature search was conducted from the following databases: PubMed, Scopus and Google Scholar. The search strategy was designed to identify relevant studies published up to February 2025. The search strategy included the use of keywords such as ‘medicinal plants’, ‘cervical cancer’, ‘drug resistance’, ‘phytochemicals’, ‘anticancer drugs’ and ‘bioavailability’. The Boolean operators used included AND and OR to refine the search results. The reference lists of key articles were also manually screened to identify additional relevant studies.

### 2.3. Language Restrictions

Only articles published in English were considered for review. No lower limit was placed on the publication year, allowing for the inclusion of both foundational studies and recent advances.

### 2.4. Inclusion Criteria

Studies were included if they focused on the role of medicinal plants or their phytochemicals in overcoming drug resistance in cervical cancer, investigated the molecular mechanisms underlying drug resistance, and considered the potential of using medicinal plants to enhance tumour cell response to chemotherapy.

### 2.5. Exclusion Criteria

Studies that did not focus on drug resistance in cervical cancer were excluded. Furthermore, studies lacking sufficient methodological details or experimental evidence, as well as those that were case reports or opinions without original data, were excluded.

### 2.6. Screening and Selection

**Figure 1:**
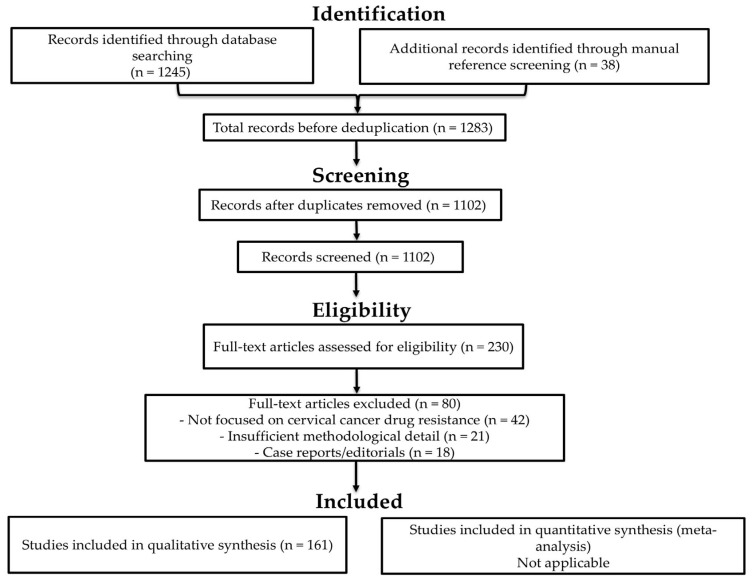
PRISMA 2020 flow diagram illustrating the study selection process.

Titles and abstracts were screened by the lead author (T.P.M.), using predefined inclusion and exclusion criteria. Other co-authors subsequently cross-checked screening decisions to ensure consistency and accuracy. All retrieved records were compiled into a Microsoft Word document and sorted alphabetically by author’s name. Duplicates were identified manually and removed, with verification against publication year and title to ensure accuracy. Full-text articles were then retrieved and assessed for eligibility. Each study was evaluated for methodological detail, relevance to drug resistance mechanisms, and reporting quality. Studies lacking sufficient experimental or mechanistic data were excluded. Although primary screening was conducted by a single reviewer, cross-checking by co-authors provided an additional layer of verification to enhance consistency and reduce the likelihood of oversight. This approach was adopted due to resource constraints but was supported by standardised criteria and documentation to maintain methodological rigour. A PRISMA 2020 flow diagram summarising the screening and selection process is provided in Figure 1. This includes the number of records identified, screened, excluded, and included in the final synthesis.

### 2.7. Data Extraction and Synthesis

Data extraction was performed using a structured template developed before the review. For each eligible study, information was collected on the study design, experimental model (in vitro, in vivo, or clinical), the medicinal plant or phytochemical investigated, and the specific mechanism of drug resistance addressed. Outcomes related to chemosensitisation, reversal of resistance, or enhancement of therapeutic efficacy were systematically recorded. Where available, details on dosage, bioavailability, and molecular pathways were noted to facilitate comparison across studies. Extraction was conducted independently by two reviewers to minimise bias, and discrepancies were resolved through discussion or consultation with a third reviewer. The extracted data were subsequently synthesised narratively, with findings grouped according to the resistance mechanism targeted (e.g., oxidative stress, epithelial–mesenchymal transition, epigenetic modulation) and mapped to the corresponding phytochemical interventions.

## 3. Processes Underlying Multidrug Resistance

**Figure 2:**
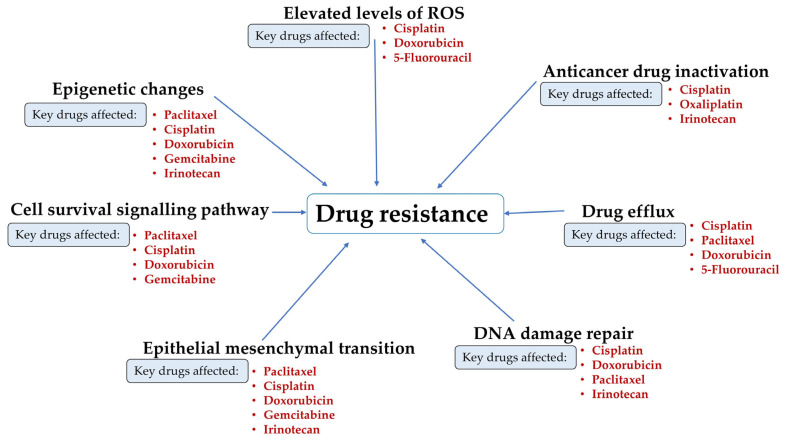
Mechanisms driving drug resistance in cervical cancer. Processes such as ROS elevation, epigenetic changes, signalling dysregulation, EMT, enhanced DNA repair, drug efflux, and drug inactivation reduce the efficacy of drugs, including cisplatin, doxorubicin, 5-fluorouracil, icaritin, vincristine, irinotecan, gemcitabine, and paclitaxel.

Due to unsuccessful therapies and advancement of the disease, drug resistance is accountable for approximately 90% of cancer-related fatalities, including those from cervical cancer [5]. Several mechanisms contribute to drug resistance, including drug efflux [12], epigenetic changes [13], elevated amounts of reactive oxygen species (ROS) [14], enhanced DNA damage repair mechanisms [15], epithelial–mesenchymal transition (EMT), anticancer drug inactivation [16], and cell survival signalling pathways [17]. Various anticancer drugs that are used in cervical cancer treatment, such as cisplatin, doxorubicin, 5-fluorouracil (5-FU), icaritin, vincristine, irinotecan, gemcitabine, and paclitaxel, are significantly impacted by each of these processes (Figure 2). This results in reduced effectiveness of the drug delivered, making cancer treatment more challenging [18].

Drug resistance can be categorised as either innate (intrinsic) or acquired, depending on the timing of its onset. Intrinsic resistance emerges in a cluster of cancerous cells before drug administration and reduces the effectiveness of the treatment. In contrast, acquired resistance develops when an anticancer agent loses its effectiveness following repeated drug administrations [5,19,20].

### 3.1. Intrinsic Drug Resistance

Some of the characteristics associated with intrinsic drug resistance include drug deterioration; altered drug receptors leading to unresponsiveness of tumour cells towards a specific treatment and signalling pathways, due to weak affinity of the drug to its molecular targets; and rapid progression of the tumour cells resulting from preceding mutations or other cellular activities associated with clusters of tumour cells [6,21]. Various mechanisms contribute to intrinsic drug resistance, such as enhanced cell survival signalling pathways, EMT, and elevated levels of ROS [22].

#### 3.1.1. Evidence Scope

The majority of studies discussed in this section are based on in vitro or animal models. While these findings provide valuable mechanistic insights and identify potential therapeutic targets, they should be interpreted as preclinical evidence unless otherwise supported by clinical trial data.

#### 3.1.2. ROS Induces Intrinsic Drug Resistance

ROS are oxygen-containing, unstable compounds such as superoxide (O_2_^−^*), hydrogen peroxide (H_2_O_2_) and hydroxyl radicals (HO*) [23]. Elevated levels of ROS in cancer cells drive DNA damage [24], genetic instability [25], and pro-tumourigenic signalling [26]. Aerobic respiration produces ROS largely via the electron transport chain in the mitochondria and ß-oxidation of fatty acids in the peroxisomes [27,28]. In some cancers, electron transport chain activation contributes to drug resistance. In cervical tumour cells, electron leakage occurring during oxidative phosphorylation triggers the production of ROS and other radicals, leading to oxidative DNA damage of the uterine epithelial cells [29]. Furthermore, elevated levels of ROS promote the triggering of cellular receptor tyrosine kinase (RTK) signalling, leading to upregulation of cell survival processes such as MAPK, PI3K/AKT, and JAK/STAT, which induce intrinsic drug resistance [30,31]. Additionally, CaSki cancer cells exposed to cisplatin activate EphA4 (Ephrin type-A receptor 4) receptor tyrosine kinase signalling via the production of reactive oxygen species, thus inducing chemoresistance [32]. Some of the ROS production is mediated by oxidoreductase enzymes such as lipoxygenase, cyclooxygenases (COXs), and NADPH oxidases (NOXs), as well as external stimuli such as UV radiation and host defence mechanisms [33].

**Figure 3:**
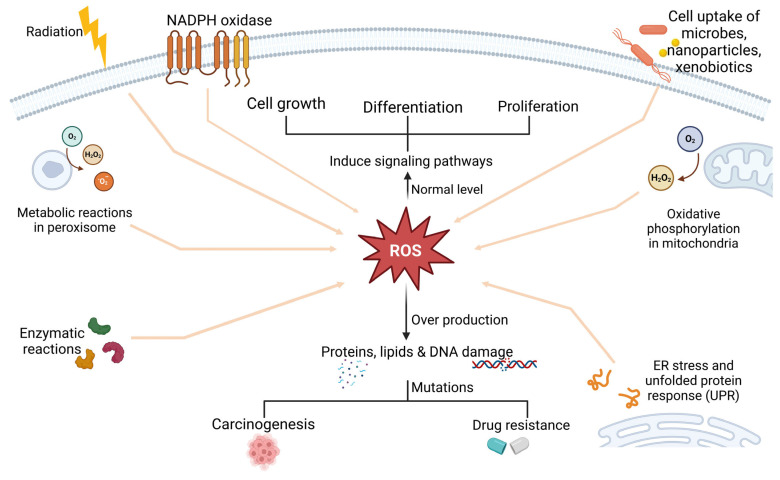
Reactive oxygen species (ROS) arise from enzymatic reactions, peroxisomal metabolism, external stimuli such as radiation or microbial invasion, mitochondrial oxidative phosphorylation, and ER stress-induced UPR. Low ROS levels regulate pathways involved in cell proliferation and differentiation, whereas elevated ROS promote carcinogenesis and resistance to anticancer therapy. This figure was created in BioRender.com.

Minimal ROS levels modulate proliferation and differentiation in non-cancerous and cancerous cells. For instance, low-to-moderate amounts of ROS under physiological circumstances interact with macromolecules through reversible oxidative changes, and this process is essential for cell growth [34]. Elevated ROS levels are associated with anticancer drug resistance, as they promote tumourigenesis and metastasis during or after treatment. Cervical cancer is mainly attributed to infections caused by the human papillomavirus (HPV). The insertion of HPV into the cellular genome causes oncoprotein expression, which is linked to persistent inflammation and increased ROS production [35]. Elevated intracellular ROS can disrupt lipids, proteins, and DNA, thus causing mutations that increase carcinogenesis in non-cancerous cells or multidrug resistance in cancer cells, as demonstrated in Figure 3 [14,36]. The interaction of ROS with proteins such as Heat Shock Protein 70 (HSP70) has been the subject of several studies [37]. For instance, ROS can increase the expression of HSP70 to provide a cytoprotective effect. In non-cancerous cells, expression of HSP70 effectively induces their response to external stresses and prevents cell death; however, when HSP70 is overexpressed in tumour cells, resistance to treatment intensifies [38,39]. Most cancer cells may survive apoptosis and develop resistance to various chemotherapeutic treatments when they are exposed to intrinsic oxidative stress. A range of chemotherapy medications utilised for treating cervical cancer may be rendered much less effective if ROS levels are elevated [40]. One common chemotherapy drug for cervical cancer is cisplatin. Elevated amounts of ROS can promote cellular processes that repair DNA damage, which can lead to resistance and eventually reduce the efficacy of cisplatin [41]. One way that doxorubicin induces cell death is by generating ROS. Conversely, tumour cells can become resistant to high ROS environments by upregulating antioxidant defences or strengthening DNA repair mechanisms, among other strategies. These changes can greatly minimise the cytotoxic effects of doxorubicin. Another chemotherapy drug that is susceptible to changes in ROS levels is 5-FU. Elevated levels of ROS in cancer cells may improve repair systems and help the cells to withstand 5-FU treatment, leading to drug resistance [11,42].

**Figure 4:**
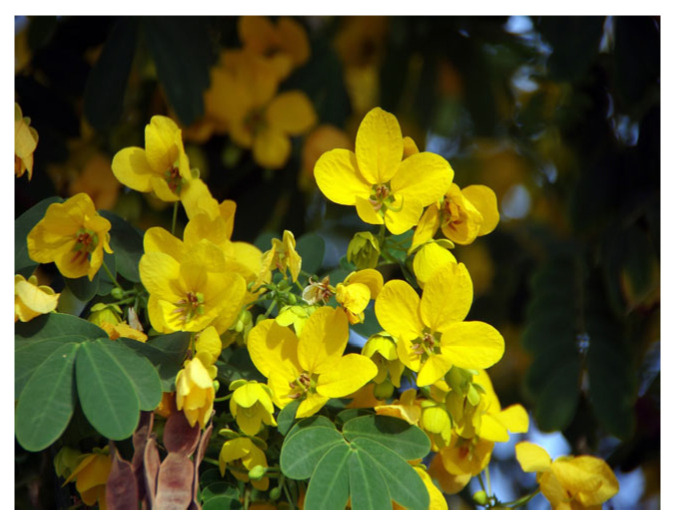
*Cassia tora* plant with flowers: The plant contains bioactive compounds such as emodin, alaternin and nor-rubrofusarin glucose that induce apoptosis and mitigate ROS. This image was adopted from https://pixabay.com/photos/flower-yellow-petals-cassia-5474624/ (accessed on 12 January 2026) by DEZALB.

Several medicinal plants have been shown to lower ROS levels, perhaps reversing multidrug resistance or making cervical cancer cells more sensitive to anticancer drugs. For instance, *Cassia tora* (Figure 4) contains emodin (an anthraquinone) that has demonstrated substantial antiproliferative effects against HeLa cells, inducing apoptosis and lowering DNA content through its antioxidant capabilities, which help to mitigate oxidative stress [43]. This process is linked to the cleavage of poly [adenosine 5′-diphosphate (ADP)-ribose] polymerase and the activation of caspase-9.

Moreover, emodin also leads to cell cycle arrest at the G2/M phase and inhibits cell growth and differentiation by downregulating cyclin A and cyclin-dependent kinase 2 (CDK2) [44,45]. Peroxynitrite (ONOO^−^) is a ROS produced when superoxide anion radicals (O^2−^) react with nitric oxide (NO) [46]. In the study conducted by Jain and Patil (2010) [47], alaternin and nor-rubrofusarin glucose (phenolic active compounds) extracted from *Cassia tora* demonstrated potent ONOO^−^ scavenging activity and led to a decrease in ONOO^−^ mediated nitration of tyrosine through electron donation.

**Figure 5:**
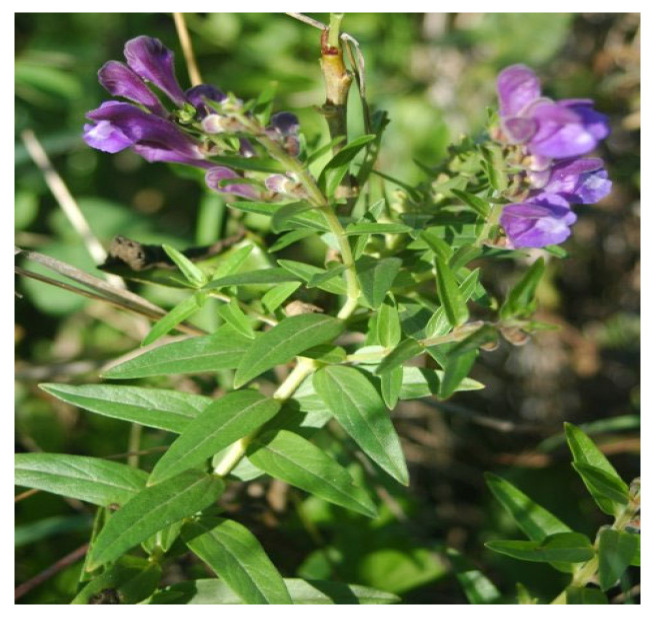
*Scutellaria baicalensis* plant: It contains a flavonoid called baicalein that demonstrated a chemosensitising effect in cervical cancer cells to cisplatin and induced apoptosis and cuproptosis. This image was adapted from Wikipedia, https://en.wikipedia.org/wiki/Scutellaria_baicalensis (accessed on 12 January 2026).

Another interesting medicinal plant is *Scutellaria baicalensis* (Figure 5), whose flavonoid constituents, such as baicalein, targeted cyclin D1 and regulated the Wnt/β-catenin signalling cascade, suppressing cell proliferation and triggering apoptosis [48].

In another preclinical study conducted by Jin et al. [49], baicalein enhanced the sensitivity of cervical cancer cells to cisplatin by inducing cuproptosis, a regulated form of cell death. In vivo experiments demonstrated that the combination of baicalein and cisplatin exerted a synergistic effect, promoting apoptosis and suppressing tumour progression. This potentiation of cisplatin cytotoxicity was mediated via the Akt signalling pathway, suggesting that baicalein may serve as a promising adjuvant for overcoming cisplatin resistance in cervical cancer therapy. In addition to the role of medicinal plants in regulating ROS levels, it is crucial to consider the impact of cell survival pathways on the efficacy of anticancer drugs.

#### 3.1.3. Cell Survival Pathways and Their Contribution to Drug Resistance

The efficiency of chemotherapeutics is greatly impacted by cell survival pathways, which promote cell proliferation and interfere with apoptosis, thereby contributing to intrinsic drug resistance. The transforming Growth Factor Beta (TGF-β) cascade regulates several cellular activities, affecting the tumour microenvironment and cancer development. It is well recognised that TGF-β may promote tumour growth in more advanced cancers or suppress tumours in early stages. It suppresses immune surveillance while concurrently promoting cell proliferation and metastasis [50]. Tumours can evade treatment because of the pleiotropic nature of TGF-β signalling, leading to resistance to drugs [51].

The antioxidant pathway in cervical epithelial tissue is governed by the transcription factor Nuclear Factor Erythroid 2-related Factor 2 (NRF2), which controls gene transcription and regulates free radicals. Factors such as oxidative stress trigger the expression of *p21* and *NRF2* genes, enhancing glutathione metabolism and thereby reducing the efficiency of anticancer drugs [52]. Under normal conditions, Kelch-like ECH-associated protein 1 (Keap1) interacts with NRF2, promoting its proteasomal degradation and thereby reducing its concentration. However, during oxidative stress, Keap1-NRF2 interaction weakens, forcing them to dissociate and sequester NRF2 in the nucleus. This process stimulates the transcription of genes responsible for cytoprotection and metabolic processes. In tumour cells, the degradation of NRF2 is often disrupted, resulting in its overexpression and accumulation, which can trigger drug resistance [17].

Moreover, abnormal oncogenes alter pathways including MAPK/JNK, Ras/Raf/ERK, PI3K/AKT, and JAK/STAT, further establishing drug resistance [53]. The PI3K/AKT/mTOR and the JAK/STAT survival pathways play roles in different biological processes such as cell survival, apoptosis, tumour development, and cell proliferation [54]. These signalling pathways are activated by HPV infection and E6/E7 expression, which disrupts several cellular and molecular processes to promote tumourigenesis. Activation of the PI3K/AKT/mTOR pathway impacts the efficacy of various anticancer drugs through various mechanisms [5,55]. For instance, when AKT is activated, it suppresses *p53* activity, thereby decreasing the apoptotic response to cisplatin therapy [56]. Additionally, activation of the PI3K/AKT/mTOR signalling cascade results in the overproduction of BCL-2, diminishing the efficacy of doxorubicin, thus contributing to doxorubicin resistance [57]. Resistance to gemcitabine has been associated with overexpression of the Ras/Raf/ERK and PI3K/AKT signalling pathways. These pathways interact and increase the expression of genes that prevent drug-induced apoptosis while also promoting cell survival and proliferation [48,58]. Furthermore, paclitaxel resistance can be triggered by transcription factors belonging to the Fox family. These factors regulate proteins such as BH3-interacting domain death agonist (Bid) and express anti-apoptotic proteins like BCL-2, initiating pathways such as MAPK/JNK that promote cell survival [59].

**Figure 6:**
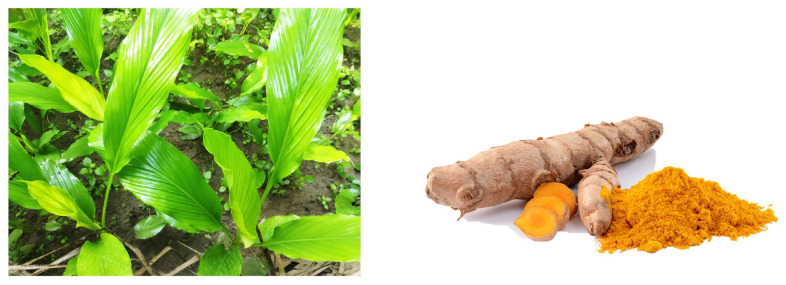
*Curcuma longa* plant and root: It contains curcumin, which has demonstrated the ability to counteract resistance to chemotherapy. These images were adopted from https://unsplash.com/s/photos/curcuma-longa (accessed on 12 January 2026) and https://en.wikipedia.org/wiki/File:Curcuma_longa_roots.jpg (accessed on 12 January 2026).

Numerous plant extracts have been shown to affect the TGF-β pathway, which can be very important for treating cervical cancer. Studies have demonstrated that some plant extracts can either stimulate or inhibit TGF-β activity, thereby influencing the susceptibility of tumours to drugs. For instance, *Curcuma longa* (Figure 6) extract (curcumin) and emodin have been shown to lower the expression of TGF-β receptor II and related Smad proteins, thus limiting tumour metastasis and invasion [60].

**Figure 7:**
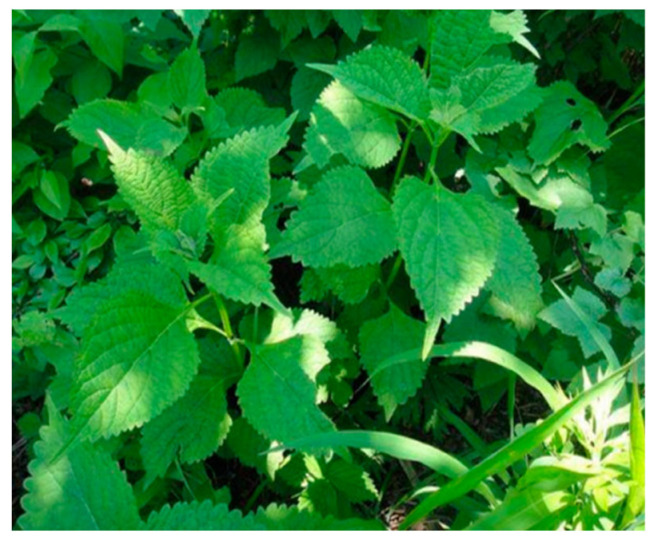
*Rabdosia rubescens* plant: It contains phytocompounds such as diosgenin and oridonin that have been demonstrated to suppress cervical cancer growth via PI3K/AKT and MAPK signalling pathways in preclinical models. This image was adapted from Chen et al., *Pathogens* 2023, CC BY licence [65].

Additionally, resveratrol has been linked to anticancer benefits by modulating the TGF-β pathway, enhancing the effectiveness of chemotherapeutic drugs and inhibiting tumour growth [61]. By disrupting mitochondrial membrane potential and increasing intracellular calcium levels, kaempferol (a type of flavonol) has demonstrated effectiveness in inhibiting cell growth and triggering apoptosis. Interestingly, it increases susceptibility to paclitaxel by decreasing *P*-glycoprotein activity [48,62]. Diosgenin (a saponin) and oridonin (a diterpenoid) extracted from *Rabdosia rubescens* (Figure 7) have been shown to suppress cervical cancer cell growth through numerous pathways, such as PI3K/AKT and MAPK signalling, inducing apoptosis, and lowering drug resistance mechanisms [63,64]. In addition to these pathways, the EMT is a fundamental process that also contributes to tumour progression and metastasis.

#### 3.1.4. The Role of Epithelial–Mesenchymal Transition (EMT) in Drug Resistance

Epithelial cells lose their epithelial phenotype through a physiological process known as EMT. The cells exhibit a mesenchymal phenotype, losing cadherin-mediated cell–cell adhesion and its polarity; however, they become more invasive [66]. During EMT, transcription factors Ovo-Like Zinc Finger 1 (OVOL1) and Ovo-Like Zinc Finger 2 (OVOL2) bind to the epithelial markers E-cadherin and cytokeratin, as well as junctional proteins like catenins and claudins. This interaction enhances the production of mesenchymal markers such as vimentin, N-cadherin and fibronectin [67,68]. Overexpression of EMT transcription factors such as Snail Family Transcriptional Repressor 1 (SNAI1) triggers drug resistance by regulating the synthesis of ATP-binding cassette transport proteins. Research has demonstrated that overproduction of Snail and Twist enhances the activity of ABC transporter promoters, leading to an increased synthesis of these proteins in tumour cells [69,70]. Overexpression of ABC transporters facilitates the removal of chemotherapeutic agents from cells, leading to a reduction in their intracellular levels and, as a result, contributing to resistance against drugs such as doxorubicin, cisplatin, gemcitabine, and irinotecan [70,71]. Cells undergoing EMT frequently evade apoptosis, which is critical for the efficacy of several chemotherapeutics. For instance, during EMT, BCL-2 and BCL-XL become overexpressed, allowing cells to survive during chemotherapy treatment and resulting in drug resistance against agents that induce cell death, such as gemcitabine, irinotecan and cisplatin [67,70,72].

**Figure 8:**
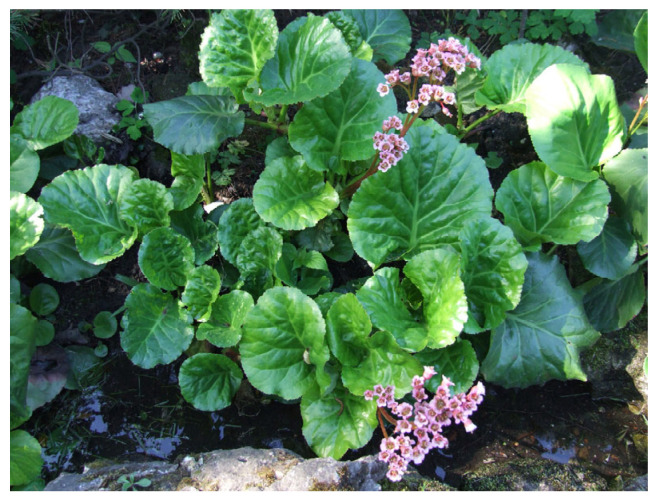
*Bergenia crassifolia*: It contains a polyphenol called bergenin that has been shown to lower the expression of angiogenic proteins such as galectin-3 and MMP-9 and reverse EMT-related drug resistance. This image was adapted from https://commons.wikimedia.org/wiki/File:Bergenia_crassifolia_a1.jpg (accessed on 12 January 2026) by Jerzy Opioła.

Numerous medicinal plants have demonstrated the ability to downregulate EMT, thereby reversing drug resistance. For instance, bergenin (a polyphenol), a phytochemical isolated from *Bergenia crassifolia* (Figure 8), has been shown to inhibit the growth of cells such as SiHa and C33A by inducing autophagy and cell death. It may also reverse EMT-related drug resistance by lowering the expression of angiogenic proteins implicated in tumour growth and metastasis, such as galectin-3 and matrix metalloproteinase-9 (MMP-9) [48,73].

Curcumin has demonstrated the ability to counteract resistance to chemotherapy by inhibiting the expression of anti-apoptotic proteins and modulating the AKT/mTOR and NF-κB signalling pathways, thereby increasing the vulnerability of tumour cells to treatments including doxorubicin and cisplatin [74,75]. Berries and grapes contain resveratrol, which suppresses regulatory proteins, including Twist and Snail, to prevent EMT and sensitise cancer cells to treatment [76]. Tumour cells can acquire resistance after being exposed to an anticancer drug.

### 3.2. Acquired Drug Resistance

The term “acquired resistance” refers to cancer drug resistance that results in tumourigenesis after the initial drug administration. Genetic factors, such as mutations, increase the emergence of acquired resistance to treatment in tumour cells by altering genes that control metabolic pathways [77]. For example, amplification of the *ATP-binding cassette subfamily B member 1* (*ABCB1*) gene, which encodes proteins associated with multidrug resistance, such as P-glycoprotein, can trigger resistance to lipophilic cytotoxic medications, including docetaxel and camptothecin, thereby reducing the chances of survival for cancer patients [78]. In genotype-matched targeted therapy, the development of acquired resistance limits the effectiveness of tumour response to treatment while also creating a significant barrier to achieving improved long-term survival outcomes [79]. Most research on acquired drug resistance has focused on understanding the molecular resistance mechanisms in cervical cancer cells. Epigenetic modification, drug efflux, and alterations in the DNA damage repair mechanism contribute to the emergence of acquired drug resistance.

#### 3.2.1. The Role of Epigenetics in Acquired Drug Resistance

Epigenetics refers to biological phenomena where phenotypic changes are passed down through generations without altering the DNA sequence. These phenomena encompass DNA methylation, acetylation, and RNA silencing. Within the context of cervical cancer, epigenetics significantly influences the emergence of drug resistance, particularly against drugs such as gemcitabine, cisplatin, doxorubicin, 5-fluorouracil, irinotecan, and paclitaxel [80]. High-risk HPV strains, including HPV-16 and HPV-18, are key factors influencing epigenetic changes in cervical cancer. The HPV oncoproteins E6 and E7 produced by these viruses alter DNA methylation patterns, accelerating tumourigenesis and promoting drug resistance. For example, E6 can disrupt the activity of p53, a crucial tumour suppressor involved in DNA damage response, permitting cells with damaged DNA to continue growing even during chemotherapeutic treatment. This interplay not only encourages carcinogenesis but also promotes drug resistance [81,82]. The synergistic interaction between viral proteins E6 and E7 and cellular resistance mechanisms significantly contributes to the increased drug resistance observed in cervical cancer cells. These viral proteins induce hypermethylation of CpG islands within tumour suppressor gene promoter regions, thereby silencing their expression, while concurrently promoting hypomethylation that activates oncogenes. Viral proteins extensively alter the transcriptional capacity of infected cells by influencing chromatin-remodelling proteins and histone modifications. These alterations not only play a crucial role in carcinogenesis but also enhance the ability of cancer cells to evade therapeutic interventions, ultimately contributing to drug resistance [83,84].

Chemotherapy resistance in cervical cancer is also influenced by factors such as Toll-like receptors (TLRs), particularly TLR4. In HPV-positive cervical cancer cells, TLR4 activation is associated with increased cell survival and proliferation, which modulates the expression of genes associated with cell cycle control and apoptosis [85]. Studies show that TLR4 expression levels are often greater in HPV-positive cells than in HPV-negative cells, particularly in cervical cancer. This enhanced expression may result in higher cell survival and proliferation, potentially promoting chemoresistance [86].

DNA methylation, which suppresses transcription processes, is a key mechanism underlying drug resistance. For instance, DNA methylation of the *Caspase 8 Associated Protein 2* (*Casp8AP2*) gene in cervical cancer reduces its expression and induces cisplatin and taxol resistance. Moreover, genetic research has uncovered that hypermethylation of *DAPK* is linked to drug resistance in various malignancies [87]. Essential tumour suppressor genes, such as *CDKN2A*, are commonly found to be hypermethylated, thereby disrupting cellular mechanisms such as cell cycle control and apoptosis, allowing tumour cells to survive doxorubicin therapy. Acetylation and methylation of histones play an important role in modulating the expression of drug resistance genes. Genes involved in apoptosis or drug sensitivity may be suppressed as a result of histone deacetylase (HDAC) dysregulation [81,88,89]. Furthermore, histone modifications can alter chromatin structure, leading to increased transcription of DNA repair genes such as *Xeroderma Pigmentosum Group F (XPF)* and *Excision Repair Cross-Complementation Group 1* (*ERCC1*), which repair cisplatin-induced DNA damage, thereby promoting cancer survival and increasing resistance to cisplatin during treatment [90]. DNA demethylation in CpGs of promoter sequences, along with histone methylation, can trigger *NRF2* expression. For instance, DNA methylation and histone methylation status in the context of oxidative stress induced by 5-FU influence the expression of *NRF2*, which then induces drug resistance to 5-FU [91]. Epigenetic modifications that enhance topoisomerase II expression or affect its activity have been shown to induce resistance to irinotecan by decreasing its ability to generate DNA breaks [90,92]. Resistance to paclitaxel has been associated with epigenetic changes that increase the expression of proteins that are responsible for microtubule stability and dynamics. Additionally, epigenetic changes can modify the expression of enzymes that regulate nucleotide metabolism, impacting gemcitabine’s activation and efficiency [20,88].

Alternative splicing is a molecular mechanism that allows the synthesis of a variety of mature mRNAs from a single gene [93]. Defects occurring in the process result in the synthesis of non-functional or unfavourable products that may initiate cancer development and progression. Drug resistance against anticancer agents may develop due to defects in the splicing process. BCL-2–interacting mediator of cell death (BIM) is a group of proteins involved in essential biological processes such as maintaining haematopoietic balance, restriction of autoimmune diseases, and initiation of cancer. When the BIM proteins are upregulated, inhibitors of cell death pathways such as cellular inhibitor of apoptosis proteins (cIAP1 and cIAP2), survivin, X-linked inhibitor of apoptosis protein (XIAP), and livin accumulate in the tumours, promoting drug resistance [94,95].

**Figure 9:**
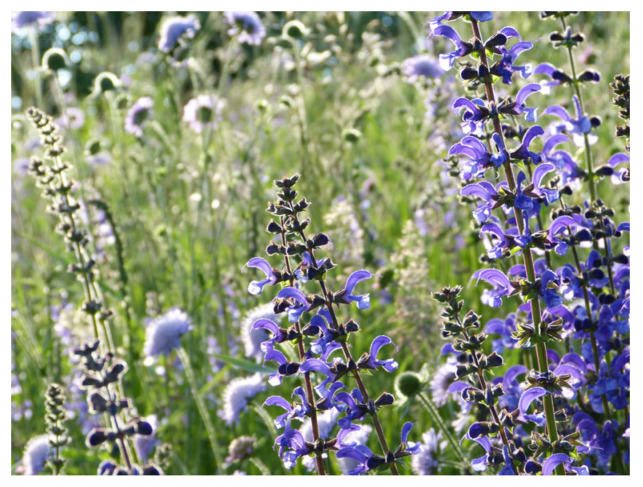
*Salvia miltiorrhiza* flowers: they contain salvianolic acid B and tanshinone IIA, which have the potential to reverse abnormal epigenetic changes. This image was adopted from https://pixabay.com/photos/salvia-white-sage-bloom-meadow-4262870/ (accessed on 12 January 2026) by silviarita.

Numerous medicinal plants and their phytochemicals have been shown to affect epigenetic modifications in preclinical models, which can be very important for treating cervical cancer. For instance, *Salvia miltiorrhiza* (Figure 9) has been associated with changes in cytosine DNA methylation and the control of secondary metabolites, which can improve its therapeutic properties. The phytochemicals in this plant, such as salvianolic acid B and tanshinone IIA, may reverse epigenetic silencing, though clinical efficacy has not yet been demonstrated [90,96].

**Figure 10:**
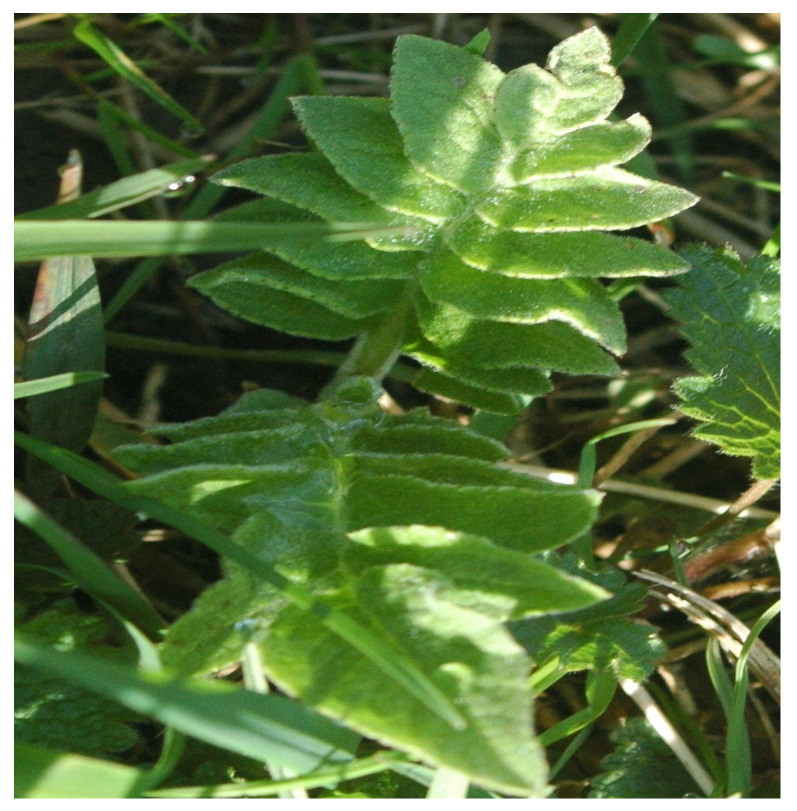
*Rhaponticum Carthamoides* plant: It contains tricaffeoylquinic acid derivatives that may regulate histone modifications and induce DNA damage. This image was adapted from https://en.wikipedia.org/wiki/Rhaponticum_carthamoides#/media/File:Rhaponticum_carthamoides.jpg (accessed on 12 January 2026) by Doronenko.

*Rhaponticum Carthamoides* (Figure 10) has demonstrated the ability to induce apoptosis in cancer cells via epigenetic pathways. The anticancer properties of its extracts, such as tricaffeoylquinic acid derivatives, may be linked to their ability to regulate histone modifications and induce DNA damage [97,98].

Through processes such as DNA methylation and histone modification, several phytochemicals interact with the epigenetic machinery, influencing gene expression. For instance, epigallocatechin-3-gallate (a type of flavonoid) has been shown to alter microRNA expression and DNA methylation, which can suppress oncogene expression and reactivate silenced tumour suppressor genes. It can modify gene expression patterns, increasing apoptosis and promoting cell cycle arrest, thereby improving chemotherapeutic efficacy and reversing resistance [99].

The most studied polyphenols, such as curcumin, quercetin, and resveratrol, have been shown to regulate both DNA methylation and histone acetylation in preclinical models. Moreover, studies have indicated that they inhibit histone deacetylases and DNA methyltransferases, leading to the downregulation of oncogenes and the reactivation of previously silenced tumour suppressor genes. This modulation can enhance the susceptibility of cervical cancer cells to chemotherapy while also overcoming drug resistance [100]. Numerous natural compounds have been identified as effective inhibitors of HPV E6 and E7 activity, which are crucial for the progression of cervical cancer. For instance, luteolin has been shown to bind to the E6 protein, preventing the degradation of p53, a vital tumour suppressor, thereby reducing the viability of HPV-infected cervical cancer cells [101]. Similarly, curcumin has demonstrated the ability to inhibit both E6 and E7 transcripts and proteins, thereby obstructing the translocation of NF-κB and AP-1 transcription factors and ultimately inducing apoptosis in HeLa, SiHa, and C33A cancer cell lines [102]. Additionally, tanshinone IIA has been reported to downregulate E6 and E7 expression while promoting apoptosis and cell cycle arrest in various cervical cancer cell lines, including HeLa, SiHa, CaSki, and C33A [103].

#### 3.2.2. Drug Efflux and Its Role in Drug Resistance

Drug efflux mechanisms have a major impact on the development of multidrug resistance. Multidrug resistance is associated with upregulated ATP-binding cassette (ABC) transporters, such as *P*-glycoprotein (P-gp), as well as members of the multidrug resistance protein (MRP) family, including MRP1 and MRP2, which are located on the tumour cell membranes and play a crucial role in mediating drug efflux [104]. *P*-glycoprotein, commonly known as ABCB1, is a chemotherapeutic drug-binding protein transcribed by *ATP-binding cassette subfamily B member 1*. The frequent administration of chemo-drugs that are ABCB1 substrates, such as doxorubicin, gemcitabine, and paclitaxel, leads to overexpression of *ABCB1*. When *ABCB1* is overexpressed, it expels chemotherapy drugs from tumour cells, decreasing their intracellular concentration and effectiveness, ultimately resulting in multidrug resistance [105,106,107]. MRP1 has been linked to a higher cisplatin efflux in cervical cancer cells. MRP1 overexpression may result in lower intracellular concentrations of cisplatin, which could reduce the cytotoxic effects of the drug [108]. MRP2 contributes to drug resistance as well; however, its expression patterns could vary. In several studies, MRP2 was found to enhance cisplatin efflux in some cancer cell lines, with some resistant cell lines exhibiting lower expression and susceptible cell lines showing greater levels [109].

Studies have shown that phytochemicals such as curcumin, resveratrol, epigallocatechin gallate, and quercetin can modify the expression and activity of ABC transporters, which can improve treatment outcomes by increasing drug retention [11]. In various cancer cell lines, curcumin, epigallocatechin gallate, and resveratrol have been shown to reduce the expression of *P*-glycoprotein (P-gp) and other ABC transporters such as MRP1. This downregulation leads to increased intracellular levels of chemotherapeutic drugs. By inhibiting P-gp, these phytochemicals can reverse MDR, thereby enhancing the sensitivity of cancer cells to treatments such as vincristine and doxorubicin [11,110]. Quercetin has been reported to inhibit several ABC transporters, including MRP, P-gp, and breast cancer resistance protein (BCRP). It can also increase the bioavailability of chemotherapy agents by preventing their efflux. This results in a greater drug accumulation within tumour cells, thereby amplifying the cytotoxic effects of drugs such as doxorubicin and cisplatin [111]. The increased cytotoxic effects of such drugs are closely linked to their ability to induce DNA damage, necessitating further exploration of how DNA damage repair induces drug resistance.

#### 3.2.3. DNA Damage Repair

When DNA damage occurs, DNA repair mechanisms are triggered to repair the damaged DNA using various repair proteins. The risk of cancer development and drug resistance increases when mutations arise in genes that code for DNA repair proteins like O-methylguanyl DNA methyltransferase (MGMT) and Ataxia telangiectasia mutated (ATM) protein kinase [112,113]. The DNA repair mechanism is precisely regulated by DNA repair-associated genes through DNA strand breaks and homologous end-joining pathways. If repair enzymes, such as DNA polymerases, are inhibited, cells containing damaged DNA may erroneously duplicate, thereby promoting tumourigenesis and increasing the chances of acquiring drug resistance [114,115]. For instance, cervical cancer cells with upregulated DNA damage repair enzyme activity could swiftly repair cisplatin-DNA adducts. Cisplatin is recognised as the most effective drug for cervical cancer treatment. It induces intra-strand DNA crosslinks that activate the nucleotide excision repair (NER) mechanism. It has been suggested that NER prevents apoptosis in cells treated with cisplatin by activating components of the ATM pathway and recruiting them to the DNA damage site. Activated ATM quickly degrades drug-DNA adducts, resulting in drug resistance [116,117]. Doxorubicin treatment forms double-strand breaks, which are normally repaired by the non-homologous end-joining (NHEJ) and homologous recombination mechanisms. Overexpression of these DNA damage response mechanisms allows cells to resist doxorubicin treatment by successfully repairing the generated damage [118]. 5-fluorouracil (5-FU) disrupts DNA synthesis and causes strand breaks by misincorporating uracil into the DNA. The cell’s capacity to repair this damage may affect the efficacy of 5-FU; hence, improved repair mechanisms may induce resistance to 5-FU [119]. Irinotecan induces DNA damage predominantly by inhibiting topoisomerase I, leading to single-stranded breaks repaired through the base excision repair pathway. Tumours with strong DNA damage response capacities are more likely to survive irinotecan treatment because their lesions can be repaired effectively [120].

Numerous medicinal plants have demonstrated potential in modulating DNA damage repair mechanisms in cancer cells, which could improve chemotherapy efficacy and help combat drug resistance. For example, curcumin has been shown to impact several processes, such as non-homologous end joining and base excision repair, which lowers the accumulation of mutations and sensitises cancer cells to chemotherapy. Furthermore, it can cause cancer cells to undergo apoptosis, which increases their vulnerability to therapies such as doxorubicin and cisplatin [121]. Additionally, resveratrol is known to modify various DNA repair mechanisms, such as homologous recombination and mismatch repair [122]. In addition to DNA damage repair, tumour cells could resist chemotherapeutic agents due to inactivation of the anticancer drug.

#### 3.2.4. Anticancer Drug Inactivation

The interactions between the drug and various cellular proteins may reduce their efficiency and potency, thereby inactivating them [123]. For instance, protein-bound cisplatin has far lower absorption into cells and tissues than free cisplatin and is considerably less cytotoxic. Plasma proteins, particularly those with thiol groups such as human serum albumin, are irreversibly bound to cisplatin, inactivating it, reducing its efficacy and leading to cisplatin resistance. The Phase II glucuronidation pathway, responsible for the detoxification of various drugs, is one of the processes involved in drug inactivation. In the Glucuronidation pathway, various types of uridine diphosphate glucuronosyltransferase catalyse the transfer of uridine-5′-diphospho-α-D-glucuronic acid to the substrate, increasing its solubility and facilitating its degradation. Overexpression of the glucuronidation pathway results in the degradation of anti-tumour drugs, thereby inactivating them [16,124,125]. One typical detoxifying mechanism is glutathione (GSH) conjugation catalysed by glutathione S-transferases (GSTs). When platinum-based drugs such as cisplatin and oxaliplatin are conjugated with GSH, they become substrates for ATP-binding cassette (ABC) transporters. This process induces resistance by drastically lowering the intracellular concentration of these drugs [8,125]. Cytochrome P450 3A4 (CYP3A4) plays a role in drug metabolism. When CYP3A4 enzymes are high, they metabolise drugs such as irinotecan into inactive metabolites, reducing the levels of SN-38 (the active form of irinotecan), compromising treatment efficacy, and leading to drug resistance [126,127].

**Figure 11:**
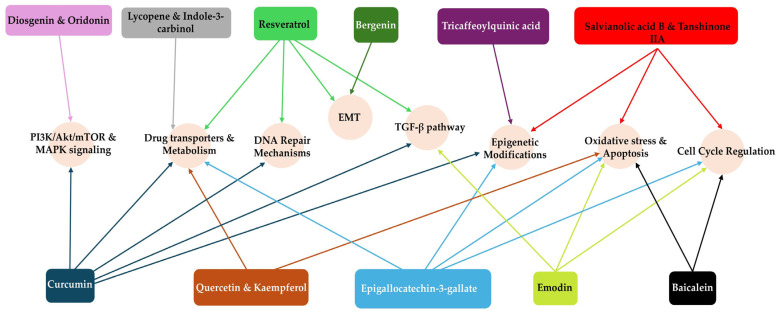
Phytochemicals and their targeted pathways: The diagram shows phytochemicals acting on major molecular processes—oxidative stress, apoptosis, cell cycle, TGF-β, DNA repair, PI3K/Akt/mTOR, MAPK, epigenetic regulation, EMT, and drug transport and metabolism. Colour-coded arrows indicate activation, inhibition, or modulation. Detailed mechanisms are provided in Table 1.

Numerous phytochemicals have been reported to reverse drug resistance mechanisms in tumour cells, especially by focusing on pathways related to drug inactivation. For instance, curcumin and quercetin improve the efficacy of certain chemotherapy drugs such as cisplatin and doxorubicin by regulating mitochondrial activities. They have been shown to reduce the activity of cytochrome P450 enzymes, which are involved in the metabolism of several anticancer drugs, enhancing their bioavailability and inducing apoptosis [128,129]. Certain phytochemicals, including epigallocatechin gallate, resveratrol, lycopene, and indole-3-carbinol, play a role in regulating GSTs and causing cancer cells to undergo apoptosis. These phytochemicals may improve chemotherapeutic efficacy by decreasing GST activity while simultaneously activating apoptotic pathways that result in cancer cell death. Integrating such phytochemicals into cancer treatments might enhance therapeutic outcomes by overcoming drug resistance mechanisms induced by excessive GST levels [44,130,131]. The summary of medicinal plants/phytochemicals and their target mechanisms is provided in Table 1 and Figure 11.

## 4. Critical Synthesis of Findings

### 4.1. Comparative Analysis of Mechanisms

Among the mechanisms identified in the reviewed studies, drug efflux mediated by ABC transporters and the activation of survival signalling pathways, including PI3K/AKT/mTOR and JAK/STAT, were the most commonly reported. While efflux is consistently linked to reduced intracellular drug accumulation, its translational relevance is limited by the absence of clinically approved inhibitors with acceptable toxicity. In contrast, EMT and epigenetic modulation, though less frequently studied, appear more promising for clinical translation because they are reversible processes and can be targeted by phytochemicals with established safety records, such as curcumin and resveratrol. Therefore, these mechanisms vary not only in their frequency of occurrence but also in their potential utility for therapeutic intervention.

### 4.2. Contradictions and Limitations Across Studies

Several contradictions emerged across the literature. For example, quercetin was reported as a potent chemosensitiser in some studies, while others found minimal or no effect, likely due to differences in cell line models, dosing regimens, and bioavailability. Similarly, the modulation of reactive oxygen species (ROS) is described variably as either protective or sensitising, contingent upon the experimental context. This variability underscores the necessity for standardised assays and more clearly defined mechanistic endpoints.

Notable limitations across the reviewed studies include a predominance of in vitro models, inconsistent documentation of phytochemical concentrations, and a lack of pharmacokinetic validation. Numerous studies utilised supraphysiological doses without adequate justification or failed to disclose formulation strategies aimed at enhancing solubility and absorption. Mechanistic assertions were frequently based on single biomarkers without corroborative evidence from upstream or downstream pathways or rescue experiments. Furthermore, few studies incorporated in vivo validation, and those that did rarely included pharmacokinetic profiling, toxicity assessment, or clinically relevant dosing. The absence of standardised protocols, inconsistent use of controls, and limited reporting of negative results suggest potential publication bias and hinder reproducibility. Collectively, these inconsistencies emphasise the need for cautious interpretation of existing data and highlight the imperative for harmonised methodologies to facilitate translational advancement.

### 4.3. Mechanisms Exhibiting Potential for Clinical Translation

From a translational research standpoint, the reversal of epithelial–mesenchymal transition (EMT) and the modulation of epigenetic processes emerge as particularly promising therapeutic strategies. Phytochemicals such as curcumin, resveratrol, and bergenin demonstrated dual functionality by targeting EMT-associated markers and concurrently downregulating efflux transporters in preclinical models. Epigenetic modulators have shown potential in restoring chemosensitivity through the reversal of HPV-induced gene silencing; however, their clinical implementation necessitates the development of enhanced delivery systems to address challenges related to limited bioavailability. Additionally, inhibitors of the PI3K/AKT/mTOR signalling pathway represent a viable avenue for intervention, although comprehensive in vivo validation remains essential.

## 5. Anticancer Potential of Medicinal Plants and Clinical Trial Issues

### Evidence Transition

While Section 3 and Section 4 primarily synthesised findings from in vitro and animal models, clinical evidence for phytochemicals in cervical cancer remains limited. Most reported effects should therefore be interpreted as preclinical insights rather than established therapeutic efficacy. This section highlights the few available clinical studies, discusses barriers to translation, and outlines the challenges of moving from mechanistic promise to patient benefit.

In addition to anticancer drug resistance, the non-specificity of anticancer drugs that induce apoptosis has been reported in several studies. Medicinal plants have been shown to interact with multiple resistance mechanisms since they contain a variety of phytocomponents. Therefore, compared to single conventional drugs, medicinal plants could offer complementary strategies for addressing chemoresistance, particularly in preclinical models. It is suggested that these compounds may offer benefits, including consistent availability, ensuring therapeutic effects, and fewer side effects [132,133,134]. While medicinal plants show promising preclinical activity, their safety and specificity in humans remain to be validated through rigorous clinical trials [135]. Plants can synthesise substances that are challenging to synthesise by chemical synthesis, rendering them a valuable source for the formulation of innovative anticancer agents that may specifically target cancer cells or sensitise tumour cells to chemotherapy [136]. Plants have also been shown to be therapeutic and efficient in extending patient survival rates, minimising chemotherapy side effects and chemoresistance, and increasing cancer patients’ quality of life, although further trials are needed to confirm effects on patient survival [137]. As a result, implementing a therapeutic approach that utilises medicinal plants could offer a strategy that inhibits cancer cell proliferation while reversing drug resistance. More focus has been placed on using medicinal plants as innovative, independent anticancer medications or in combination with already-approved anticancer agents to circumvent resistance processes to effectively treat cancer. Some clinical studies suggest that integrating phytochemicals with conventional chemotherapy can improve patient outcomes. For instance, curcumin has demonstrated improved efficacy when combined with gemcitabine [129]. Despite this potential, the number of clinical trials remains limited compared to preclinical studies. Phytochemicals can synergise with traditional chemotherapeutic agents, potentially increasing their effectiveness while reducing toxicity. This synergy is supported by various studies, showing that combinations of phytochemicals may induce cell death and inhibit tumour growth in preclinical models [138,139,140].

Although several studies demonstrate the anticancer potential of medicinal plants, only a few of the phytochemicals reviewed have documented human clinical trials (Table 2); most remain supported by preclinical evidence. Cervical-specific trials are scarce, with curcumin being the only compound currently under investigation in invasive cervical cancer.

The bioavailability of plant-derived compounds can be a significant challenge since many active compounds may not be easily absorbed or metabolised in the body. The body’s capacity to absorb and use these phytochemicals can be influenced by variables including solubility and stability. The water solubility of phytochemicals, particularly phenolics and flavonoids, hinders their ability to pass through intestinal lipid membranes, leading to inadequate bioavailability and absorption. For instance, curcumin has limited bioavailability since it is poorly soluble in water [48,141,142]. Furthermore, phenolic aglycone metabolism can reduce bioavailability by increasing their water solubility and facilitating excretion. Phytochemicals such as epigallocatechin gallate and resveratrol confront similar issues due to low absorption as well as fast first-pass metabolism [143,144].

Plant-derived compounds face substantial regulatory and translational barriers. Unlike conventional pharmaceuticals, phytochemicals often lack standardised formulations, leading to batch variability and inconsistent therapeutic effects. Regulatory frameworks for herbal compounds remain fragmented, delaying trial approval and market access. Funding constraints further limit trial size and duration, reducing statistical power and generalisability [145,146,147]. Additionally, many studies lack pharmacokinetic data, toxicity profiling, and reproducible formulations, impeding clinical advancement. Although some are generally considered safe, phytochemicals can become toxic at higher doses or with overexposure. For instance, a study by Liu et al. (2022) has demonstrated dose-dependent adverse reactions for curcumin [148]. The reproducibility of research findings is further confounded by differences in the chemical composition of plant extracts, which are impacted by parameters such as geographic origin as well as preparation methods. This discrepancy may result in different batches of the same plant extract exhibiting varying therapeutic effects. Furthermore, reproducibility is further impeded by inadequate reporting guidelines and methodological errors [145,149].

There is conflicting evidence regarding the safety and effectiveness of phytochemicals in humans, despite several studies suggesting their potential to overcome chemoresistance. Due to variations in patient metabolism, bioavailability, and tumour microenvironments, some research indicates that the benefits seen in vitro may not fully translate to clinical outcomes. Furthermore, different study methods and phytochemical formulations may produce different results [11,150]. Despite promising preclinical data, many phytochemicals fail to advance clinically due to poor solubility, rapid metabolism, and low tissue penetration. Without formulation strategies to improve bioavailability, therapeutic concentrations cannot be achieved in vivo [151]. Moreover, the lack of intellectual property protection for natural compounds reduces commercial incentive for pharmaceutical investment, further slowing translation [152].

**Table 2:** Clinical trial evidence for selected phytochemicals.

Phytochemical	Cervical Cancer Trial	CIN/HPV Human Study	Trials in Other Cancers	Outcome Summary	Reference
Curcumin	Recruiting trial adjunct to chemotherapy (Phase II, NCT06080841)	Capsule for CIN (Phase II, NCT02554344)	Pancreatic cancer (Phase II, NCT00192842); Breast cancer (Phase I/II, NCT01740323)	Safe; poor bioavailability; cervical trial ongoing; CIN regression signals; safe adjunct in pancreatic and breast	[153,154,155,156]
Epigallocatechin-3-gallate	None	None	Prostate cancer (Phase II, NCT00596011)	Tested as Polyphenon E in men undergoing prostatectomy; safe; showed biomarker modulation (PSA, tissue EGCG levels)	[157]
Resveratrol	None	None	Colon cancer prevention (Phase I, NCT00256334)	Safe; modest antiproliferative effects in colorectal mucosa	[158]
Diindolylmethane (Indole-3-carbinol derivative)	None	CIN regression (not formally registered)	Breast cancer prevention (Phase I, NCT01391689)	Safe; exploratory efficacy	[159]
Indole-3-carbinol (I3C)	None	CIN regression (Phase II, NCT00988845)	None	Regression in subset; safe	[160]
Active Hexose Correlated Compound	None	Persistent HPV clearance (Phase II, NCT02405533)	None	Increased HPV clearance rates; safe	[161]

## 6. Study Limitations

The current literature on the clinical efficacy and safety of natural products in overcoming drug resistance in cervical cancer is limited, with most evidence derived from preclinical studies. Well-designed clinical trials are needed to validate these findings. A major challenge is the poor bioavailability of many promising phytochemicals, which may limit their therapeutic use. Approaches such as nanoformulations, liposomes, micelles, and polymer-based delivery systems are under investigation to improve absorption, stability, and tumour-specific targeting. These strategies aim to overcome solubility barriers, protect compounds from first-pass metabolism, and enhance intracellular delivery, but further work is needed in this area. Given the scarcity of clinical data, conclusions regarding therapeutic efficacy must be interpreted cautiously. This review is limited by the availability of published clinical studies and may not capture all relevant information on the topic.

## 7. Conclusions

Drug resistance accounts for many cancer treatment failures. Regardless of efforts to combat drug resistance, there is currently no viable solution. Consequently, uncovering the underlying molecular processes of drug resistance is crucial for the development of various cervical cancer treatments. Medicinal plants may offer a complementary strategy for addressing drug resistance during cervical cancer treatment due to their diverse phytochemical profiles that show anticancer activity in preclinical models. However, these findings are primarily based on in vitro and in vivo studies, and their translation to clinical success remains uncertain. Future research should focus on well-designed clinical trials to determine the efficacy and safety of specific phytochemicals in reversing drug resistance. Given the promising anticancer properties and drug-sensitising effects observed in vitro, potential phytochemicals such as curcumin, resveratrol, and kaempferol should be further investigated in clinical trials. More studies are needed to address their bioavailability and examine nanoformulations, liposomes and other drug delivery systems that can improve the absorption, distribution, and targeted delivery of phytochemicals to the cancer cells, as well as clarify their mechanisms of action. Additionally, investigating the synergistic effect of combining phytochemicals with conventional chemotherapy drugs could enhance therapeutic efficacy while reducing toxicity, though this requires further validation and robust clinical evidence. By addressing these challenges, the therapeutic relevance of medicinal plants may be clarified, contributing to future strategies for overcoming drug resistance in cervical cancer.

## Figures and Tables

**Table 1:** Summary of medicinal plants targeting drug resistance in cervical cancer and their target mechanisms.

Medicinal Plant/Source	Active Compound(s)	Target Mechanism(s)	Study Model(s)	Evidence Type	Reference(s)
*Cassia tora*	Emodin, Alaternin, Nor-rubrofusarin glucose	Mitigate oxidative stress; Activation of caspase-9 and induction of apoptosis. Arrest cell cycle at the G2/M and downregulation of cyclin A and CDK2. Scavenge peroxynitrites. Lowering the expression of TGF-β receptor II and related Smad proteins. Induces apoptosis and reduces DNA content through its antioxidant properties.	Cervical cancer cell lines (Bu 25TK, Ca Ski, HeLa, and ME-180) C57BL/6 mice.	In vitro and in vivo	[43,44,45,47]
*Scutellaria baicalensis*	Baicalein	Target cyclin D1 and modulate the Wnt/β-catenin signalling cascade. Induce apoptosis and cuproptosis.	Cervical cancer cell lines (SiHa and C33A). In vivo (BALB/c female nude mice).	In vitro and in vivo	[48,49]
*Curcuma longa*	Curcumin	Lower the expression of TGF-β receptor II and related Smad proteins. Inhibit the expression of anti-apoptotic proteins and modulate the Akt/mTOR and NF-κB signalling pathways. Inhibit histone deacetylases and DNA methyltransferases, thus downregulating oncogenes. Impact non-homologous end joining and base excision repair. Downregulation of the expression of *P*-glycoprotein (P-gp) and other ABC transporters like MRP1. Reducing the activity of cytochrome P450 enzymes and triggering apoptosis. Inhibit E6/E7 transcripts and proteins, thus obstructing the translocation of NF-κB and AP-1 transcription factors, inducing apoptosis.	Cervical cancer cell lines (HeLa, SiHa, C33A, Ca Ski) C57BL/6 mice (In vivo)	In vitro and in vivo	[60,74,100,102,110,121,128]
Grapes, Berries	Resveratrol	Modulating the TGF-β pathway and enhancing the effectiveness of chemotherapeutic drugs. Suppressing regulatory proteins, including Twist and Snail, to prevent EMT. Regulate both DNA methylation and histone acetylation. Downregulation of the expression of *P*-glycoprotein (P-gp) and other ABC transporters like MRP1. Modifying various DNA repair mechanisms, such as homologous recombination and mismatch repair. Decreasing GST activity and activating apoptotic pathways.	MCF-7 and MCF-7/DOX cells Transgenic mice Cervical cancer cell line (HeLa)	In vitro and in vivo	[11,61,76,100,122,131]
Various plants	Kaempferol	Disrupting mitochondrial membrane potential. Increasing intracellular calcium levels. Inhibiting cell proliferation and inducing apoptosis.	Cervical cancer cell line (HeLa)	In vitro only	[48,62]
*Rabdosia rubescens*	Diosgenin, Oridonin	Targeting PI3K/AKT and MAPK signalling and suppressing cell growth.	Cervical cancer cell lines (SiHa, Ca Ski). Ovarian cancer cell line (SKOV3).	In vitro only	[63,64]
*Bergenia crassifolia*	Bergenin	Lowering the expression of angiogenic proteins such as galectin-3 and MMP-9 and reversing EMT-related drug resistance.	Cervical cancer cell lines (SiHa and C33A)	In vitro only	[48,73]
*Salvia miltiorrhiza*	Salvianolic acid B; Tanshinone IIA	Modulates epigenetic changes. Targeting cytosine DNA methylation Downregulate E6 and E7 expression, thus promoting apoptosis and cell cycle arrest.	Cervical cancer cell lines. (HeLa, SiHa, C33A, Ca Ski).	In vitro only	[90,96,103]
*Rhaponticum Carthamoides*	Tricaffeoylquinic acid	Regulate histone modifications and induction of DNA damage. Inducing apoptosis	Cells obtained from surgical specimens from a patient. Rabbit model	In vitro and in vivo	[97,98]
*Camellia sinensis*	Epigallocatechin-3-gallate	Altering microRNA expression and DNA methylation, suppressing oncogene expression. Modifying gene expression patterns, increasing apoptosis and cell cycle arrest. Decreasing the production of *P*-glycoprotein (P-gp) and other ABC transporters like MRP1. Decreasing GST activity and activating apoptotic pathways.	Cervical cancer cell lines (HeLa, Ca Ski) Endocervical adenocarcinoma cells KB-C2	In vitro only	[11,99,110,131]
Various plants	Quercetin	Inhibiting histone deacetylases and DNA methyltransferases and downregulating oncogenes Inhibiting several ABC transporters, including MRP, P-gp, and breast cancer resistance protein (BCRP). Downregulation of the activity of cytochrome P450 enzymes and inducing apoptosis	C57BL/6 mice (In vivo)	In vivo only	[11,100,111,128,129]
Various fruits and vegetables	Lycopene; Indole-3-carbinol	Decreasing GST activity and activating apoptotic pathways	Cervical cancer cell lines. (HeLa, Ca Ski). Endocervical adenocarcinoma cells KB-C2.	In vitro only	[130,131]

## Data Availability

No new data were created or analysed in this study. Data sharing is not applicable to this article.
